# Dual-Objective Optimization of G^3^-Continuous Quintic B-Spline Trajectories for Robotic Ultrasonic Testing

**DOI:** 10.3390/s25185693

**Published:** 2025-09-12

**Authors:** Pengzhi Ma, Chunguang Xu

**Affiliations:** School of Mechanical Engineering, Beijing Institute of Technology, Beijing 100081, China; xucg@bit.edu.cn

**Keywords:** ultrasonic testing, robot assisted, trajectory planning, engine blade

## Abstract

To address the challenges of unstable motion and insufficient detection accuracy in robotic scanning trajectories, particularly under high curvature and irregular shape conditions during ultrasonic testing of complex free-form surface workpieces, this paper proposes a G^3^ continuous trajectory planning and optimization method based on quintic B-spline curves. First, the scanning trajectory of the robot is represented by a parametric curve, with explicit expressions for position, velocity, acceleration, and jerk derived in the form of quintic B-splines. These expressions ensure continuity in position, velocity, acceleration, and jerk (C^3^/G^3^ continuity), thus maintaining high-order geometric continuity and motion stability of the trajectory. Second, to achieve the dual optimization objectives of trajectory smoothness and surface fitting, this paper constructs a composite objective function that incorporates both the integral of acceleration squared and the surface fitting error. The smoothness index is weighted by the sum of the square integrals of the second and third derivatives of the trajectory, thereby suppressing high-order oscillations, while the fitting index is based on the mean square error between the robot end-effector path and the target surface. Finally, a numerical optimization algorithm is utilized to solve the objective function, resulting in an optimal scanning trajectory that ensures both motion stability and fitting accuracy, while maintaining G^3^ continuity. Simulation and experimental results demonstrate that this method effectively mitigates trajectory mutations and oscillations, enabling efficient and high-precision automatic ultrasonic testing, and provides a reliable trajectory planning strategy for online non-destructive testing of complex curved workpieces.

## 1. Introduction

Ultrasonic testing represents the most extensively employed and technically mature non-destructive evaluation technique. Robotic automated ultrasonic testing has become increasingly prevalent in industrial quality inspection and maintenance, offering significant advantages over traditional manual methods [1,2,3,4,5,6,7,8,9,10,11,12]. Its automation and intelligent capabilities greatly enhance detection accuracy and operational efficiency, enabling consistent, high-quality inspections. By minimizing human involvement, this technology reduces the likelihood of operator error, ensuring more reliable results. Furthermore, its ability to handle complex, repetitive tasks efficiently contributes to a reduction in labor costs and time, providing a clear edge in industrial applications. Enhancing robotic scanning velocity and guaranteeing the precision of ultrasonic inspection have long been paramount concerns for researchers worldwide. During the scanning process, it is imperative to balance sufficiently high traversal speeds—to minimize overall inspection time—with sufficiently smooth trajectories to avert vibration-induced signal distortions, thereby maximizing scan coverage and defect-detection accuracy. Accordingly, trajectory optimization must integrate the acoustic propagation characteristics of ultrasonic waves by judiciously planning the robotic instantaneous velocity and acceleration so that the sound-beam incidence angle remains collinear with the surface normal of the workpiece, thus yielding an optimal signal-to-noise ratio. In summary, techniques such as path optimization, acceleration–deceleration control, and related trajectory-planning technologies exert a critical influence on both the speed and stability of autonomous robotic ultrasonic inspection systems.

Ultrasonic scanning trajectory planning is the most basic and core function in the robot ultrasonic testing system, and it is also the key technology to improve the scanning accuracy of the equipment, accelerate the scanning speed, and enhance the stability of the operation. Based on the geometric information of the measured component, combined with the appropriate acceleration and deceleration strategy, the technology generates the path curve with the help of computer-aided manufacturing (CAM) tools, and performs interpolation and densification on this basis, and finally obtains the control instructions that drive each motion axis. In recent years, the research of trajectory planning has mainly focused on path smoothing optimization [13,14], acceleration and deceleration strategy optimization [15], look-ahead algorithm design [16,17], and parameter curve interpolation method.

When a component’s curvature changes abruptly or its contour is discontinuous, the surface normal undergoes a sudden jump. This abrupt change induces instantaneous variations in the robot’s velocity and acceleration, leading to undesirable vibrations and impact forces. By optimizing the path, the initially discrete trajectory—comprised of successive short line segments—is transformed into a higher-order, continuous, and smooth curve, a prerequisite for the robot’s high-speed, high-precision, and high-stability operation [18,19]. Path-optimization methods are typically classified into two categories: global optimization and local optimization. Global optimization employs approximation or interpolation techniques to uniformly fit the entire trajectory. Erkorkmaz et al. employed quintic polynomial spline curves to achieve C^2^-continuous fitting of short-segment paths [20]. Bi et al. utilized double NURBS splines to perform a global fit of straight-line segment trajectories in five-axis CNC machining [21]. Local optimization introduces an alternative curve at the junction of two adjacent line segments to achieve local smoothing. Commonly used methods include Bézier curves [22], polynomial curves [23], Pythagorean Hodograph (PH) curves [24], and B-spline curves [25]. Zhao et al. employed a cubic B-spline curve with five control points to smooth the junctions between adjacent line segments, precisely controlling the fitting error by adjusting the positions of the spline’s control points [25].

Beudaert et al. [26] proposed a five-axis tool path smoothing method based on double splines, which extended the three-axis angular smoothing technique presented in [25] to five-axis cutting paths. In this approach, two cubic B-splines are employed to smooth the tool tip trajectory and the position of the fixed point on the tool shaft, respectively. Subsequently, the parameters of these two paths are coupled through a third B-spline to ensure synchronization between the position and direction. To avoid iterative procedures, Yang [13] employed a quintic B-spline to smooth the position trajectory in Cartesian space, applied an analogous smoothing to the orientation trajectory in the machine coordinate frame, and directly determined the control points by enforcing synchronization conditions and imposing error constraints. Tulsyan [14] introduced a decoupled local-smoothing approach in which the position trajectory is modeled by a quintic B-spline to guarantee G^3^ continuity, while the tool-axis orientation is independently smoothed via a normalized B-spline on the unit sphere. To synchronize position and orientation and to confine the smoothing error within prescribed bounds, the control points of the directional B-spline are refined using a Newton–Raphson iterative scheme. Shi et al. [27] applied a double Pythagorean-hodograph (PH) curve to smooth the five-axis tool path; however, within this dual-spline framework, the smoothing error in tool orientation is inherently coupled with the positional smoothing error. Ye Ding [28] introduced an analytically decoupled, C^3^-continuous local path-smoothing technique. A quintic B-spline is inserted at the vertices of the tool’s position and orientation trajectory, and its transition length is optimized to achieve a G^3^/C^3^-continuous, synchronous smooth transition in both the reference frame and rotational space. This method markedly enhances the motion smoothness and tracking accuracy of industrial robots. Its effectiveness has been demonstrated through both simulation and experimental validation.

In the presence of complex geometric features on the blade surface, trajectory optimization must reconcile the scanning path with the surface topology by ensuring high-order continuity of the motion curve. This study introduces a G^3^-continuous trajectory planning approach predicated on quintic B-splines, and presents its mathematical formulation and derivation in detail. First, the blade surface is reconstructed in three dimensions, and for each sampling point, the surface normal and curvature are computed. The scanning region is then partitioned into multiple subdomains via localized path-segmentation optimization. Within each subdomain, a quintic B-spline curve satisfying G^3^ continuity is generated, guaranteeing seamless transitions not only in position and tangential direction but also in curvature and curvature derivative. Leveraging these curves, the robot can adaptively adjust the transducer’s orientation and incidence angle in real time—guided by the instantaneous normal-vector information—to maximize acoustic–surface coupling efficiency, thereby enhancing C-scan image quality and defect-recognition accuracy in ultrasonic inspection.

## 2. Experimental Methods and Configuration

### 2.1. System Components

**Figure 1:**
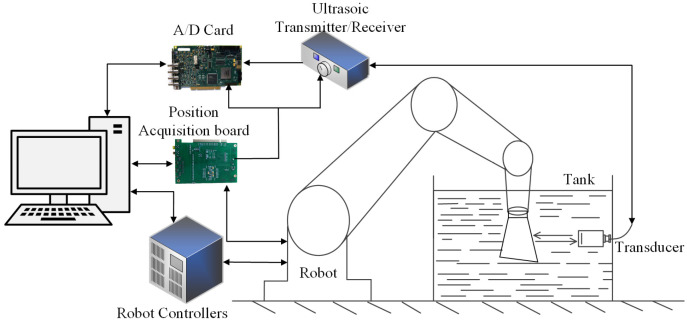
Overview of the robot-assisted UT system.

The robotic ultrasonic testing system consists of two interdependent subsystems: hardware and software. The hardware subsystem (Figure 1) comprises an industrial robot and its controller, a pulse transceiver, an ultrasonic data-acquisition module (A/D card), an ultrasonic transducer with a probe-mounting fixture, an industrial control computer, and a coupling-fluid circulation system. The software subsystem adopts a two-tier architecture: the upper tier executes global scheduling and decision-making, while the lower tier is responsible for real-time motion control and preliminary signal processing. The hardware delivers precise mechanical motion and captures raw ultrasonic signals, whereas the software interprets these signals, computes optimized scanning trajectories, and issues corresponding control commands. Together, they form a cohesive, fully automated robotic ultrasonic inspection platform.

### 2.2. Robotic Scanning Path Planning

**Figure 2:**
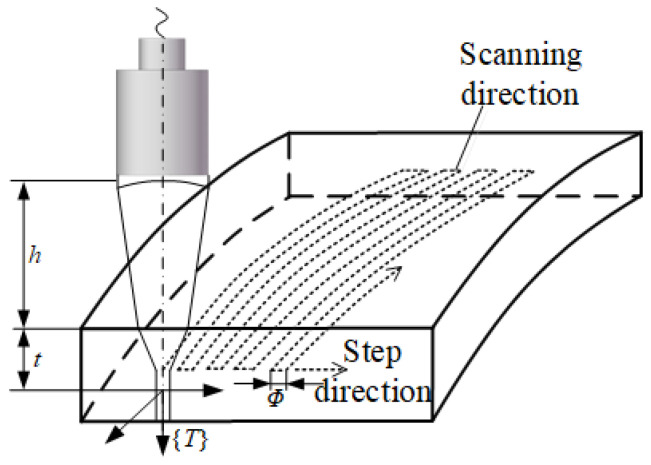
Ultrasonic transducer surface sweep path.

The robotic scanning trajectory is generated via CAD/CAM-based planning. Xiao Zhen [7] and Melo [4] have presented a comprehensive review of robotic trajectory planning, outlining a range of design and calibration techniques. In formulating the sweeping trajectory for blade inspection, several parameters must be accounted for: the ultrasonic transducer’s focal length (*F*), the focal column diameter (*Φ*), the sound velocity in the specimen material (*C_M_*), and the sound velocity in water (*C_W_*). These parameters are critical for optimizing detection sensitivity and spatial resolution. For example, when planning the focusing probe’s path for surface inspection (Figure 2), the water-path distance, h—defined as the separation between the transducer face and the inspection surface—is calculated as follows:

(1) $$h=F-t(C_{M}/C_{W})$$

In Equation (1), t represents the depth of focus, and the water range is calculated using this equation based on the specified detection depth, ensuring that the focus is aligned with the target detection depth. For the surface detection path planning shown in Figure 2, the tool coordinate system {T} is defined at the focal point. The water range is maintained at h, with the step interval approximated as half the diameter of the acoustic beam. Additionally, the incidence angle is either aligned with the normal to the surface or set at a predetermined angle to optimize coverage across the entire detection area.

### 2.3. Mathematical Modeling of Scanning Trajectory

The objective function for robot-guided ultrasonic scanning trajectory optimization is typically formulated as a weighted combination of two principal criteria, trajectory smoothness and surface conformity:

(2) $$\begin{array}{c} minJ=\alpha \int_{0}^{1}∥s^{\prime \prime }(t){∥}^{2}dt+\beta \int_{0}^{1}∥s(t)-p_{surf}(t){∥}^{2}dt \end{array}$$

$s\left(t\right)$ represents the parametric representation curve of the robot scanning trajectory;

$s^{\prime \prime }(t)$ is the acceleration of the trajectory curve (the second derivative of the curve), which is used to measure the smoothness of the trajectory;

$p_{surf}(t)$ represents the closest point to the trajectory curve on the blade surface;

α, β are the weight coefficients of smoothness and fit, respectively.

The constraint conditions include the spatial constraint and the surface fitting constraint of the trajectory:

(3) $$s.t.\left\{\begin{array}{cc} s(t)\in \Omega_{B}, & \;\forall t\in [0,\;1]\\ ∥s(t)-p_{\mathrm{surf}}(t)∥\leq \delta , & \;\forall t\in [0,\;1] \end{array}\right.$$

δ: the maximum allowable deviation between the trajectory and the blade surface (used to ensure that the robot endpoint always maintains a constant distance from the surface);

s(t): parametric trajectory curve, which is specifically selected as the quintic B-spline curve in this study;

$\Omega_{B}$: the outer envelope constraint of the robot workspace.

In order to describe the above problems in detail, the scanning trajectory in this paper is based on the quintic B-spline curve, which is defined as follows:

Let the trajectory curve be s(t), which is represented by quintic k=5 B-spline:

(4) $$s\left(t\right)=\sum_{i=0}^{n}N_{i,5}\left(t\right)P_{i},t\in \left[0,1\right]$$

s(t): the position vector of the trajectory curve on the parameter t∈[0,1];

$P=\{P_{0},P_{1},\ldots ,P_{n}\}$: the control points to be optimized;

n: the number of control points of the B-spline curve;

$N_{i,5}(t)$: a B-spline basis function, given by the Cox–de Boor recursive formula:

The specific recursive formula is:

(5) $$N_{i,0}(t)=\left\{\begin{array}{cc} 1, & \;t_{i}\leq t<t_{i+1}\\ 0, & \;\mathrm{others} \end{array}\right.$$

The higher-order basis functions are calculated by the following recursive formula:

(6) $$N_{i,k}(t)=\frac{t-t_{i}}{t_{i+k}-t_{i}}N_{i,k-1}(t)+\frac{t_{i+k+1}-t}{t_{i+k+1}-t_{i+1}}N_{i+1,k-1}(t),k=1,2,\ldots ,5$$

t is the node vector of B-spline, which is generally selected as a uniform node vector or quasi-uniform node vector.

Optimize the derivation process of the objective function. The optimization objective function consists of two parts:

(1) Smoothness term derivation:

The smoothness index of the trajectory is generally described by the acceleration square integral:

(7) $$J_{smooth}=\int_{0}^{1}∥s^{\prime \prime }(t){∥}^{2}dt$$

By solving the second derivative of the B-spline:

(8) $$s^{\prime \prime }(t)=\frac{d^{2}}{dt^{2}}\sum_{i=0}^{n}P_{i}N_{i,5}(t)=\sum_{i=0}^{n}P_{i}N_{i,5}^{\prime \prime }(t)$$

So

$$J_{smooth}=\int_{0}^{1}∥\sum_{i=0}^{n}P_{i}N_{i,5}^{\prime \prime }(t){∥}^{2}dt$$

By discretizing the integral (such as the Gauss integral or the Simpson integral), it can be approximated as:

(9) $$J_{smooth}\approx \sum_{j=1}^{M}∥s^{\prime \prime }(t_{j}){∥}^{2}\Delta t$$

$t_{j}\in [0,\;1]$ is the sampling point on the trajectory, and Δt is the integral step size.

(2) Derivation of fit term:

The trajectory fitness index is the minimum distance integral between the trajectory of the incident point of the acoustic beam and the blade surface, which is expressed as:

(10) $$J_{fit}=\int_{0}^{1}∥s(t)-p_{surf}(t){∥}^{2}dt\approx \sum_{j=1}^{M}∥s(t_{j})-p_{surf}(t_{j}){∥}^{2}\Delta t$$

Therefore, the objective function of the overall optimization problem is:

(11) $$J(P)=\alpha \sum_{j=1}^{M}∥s^{\prime \prime }(t_{j}){∥}^{2}\Delta t+\beta \sum_{j=1}^{M}∥s(t_{j})-p_{surf}(t_{j}){∥}^{2}\Delta t$$

M is the number of discrete points; Δt is the parameter step length.

Constraint conditions:

(1) Spatial outer envelope constraint:
  $$x_{min}\leq x_{i}\leq x_{max},y_{min}\leq y_{i}\leq y_{max},z_{min}\leq z_{i}\leq z_{max},i=0,\ldots ,n$$
(2) Surface distance constraint:
  (12) $$d\left(s\left(t_{j}\right),p_{surf}\left(t_{j}\right)\right)\leq \delta ,\forall j\in \left[1,M\right]$$

### 2.4. The Objective Function of Trajectory Smoothing Optimization

To achieve a high level of smoothness in robot scanning motion, the objective function for trajectory optimization is formulated as follows: The smoothness of the trajectory is primarily governed by the control of trajectory acceleration (second derivative) or even the third derivative (jerk). In this study, the integral of the square of the acceleration along the trajectory is adopted as a measure of smoothness, as outlined in references [29,30]:

(13) $$\begin{array}{c} J_{\mathrm{smooth}}(P)=\int_{0}^{1}∥s^{\prime \prime }(t){∥}^{2}dt \end{array}$$

Considering the actual numerical solution problem, the trajectory parameter t is discretized into a finite number of nodes:

(14) $$\begin{array}{c} J_{\mathrm{smooth}}(P)\approx \sum_{i=1}^{M}∥s^{\prime \prime }(t_{i}){∥}^{2}\Delta t,t_{i}\in [0,1] \end{array}$$

In order to ensure the degree of fit between the trajectory of the incident point of the ultrasonic beam and the surface of the blade, the trajectory fit item is added:

(15) $$\begin{array}{c} J_{\mathrm{fit}}=\int_{0}^{1}∥s(t)-p_{surf}(t){∥}^{2}dt\approx \sum_{i=1}^{M}∥s(t_{i})-p_{surf}(t_{i}){∥}^{2}\Delta t \end{array}$$

The final optimization objective function is expressed as:

(16) $$\begin{array}{c} J(P)=\alpha \sum_{i=1}^{M}∥s^{\prime \prime }(t_{i}){∥}^{2}\Delta t+\beta \sum_{i=1}^{M}∥s(t_{i})-p_{surf}(t_{i}){∥}^{2}\Delta t \end{array}$$

M represents the total number of discrete sampling points;

Δt is the parameter step size when integrating.

The mathematical model of scanning trajectory optimization established in this paper can be expressed as a constrained optimization problem:

(17) $$\begin{array}{c} \underset{P}{min}J(P),s.t.\{\begin{array}{c} s(t)\in \Omega_{B}\\ ∥s(t)-p_{surf}(t)∥\leq \delta \end{array} \end{array}$$

P denotes the set of control points of a B-spline.

$\Omega_{B}$ is an outer envelope constraint framework.

δ is the maximum allowable value of the distance between the trajectory and the surface.

### 2.5. Ultrasonic C-Scan Trajectory Optimization Strategy

In robot-assisted ultrasonic testing technology, trajectory optimization plays a crucial role in ensuring that the robot performs ultrasonic scanning tasks both efficiently and accurately. This is particularly critical when dealing with complex surface workpieces, such as blades and other free-form surfaces characterized by high curvature variations and irregular shapes. The smoothness and stability of the trajectory directly influence the stability, motion efficiency, and ultrasonic detection accuracy of the robot’s movement.

During the robot’s ultrasonic scanning process, the smoothness of the trajectory is reflected in the continuity of each derivative of the trajectory curve. In the domain of robot motion planning, it is generally required that the trajectory satisfies at least G^3^ continuity, ensuring the continuity of position, velocity, acceleration, and jerk (C^3^ or G^3^ continuity), in order to achieve higher motion stability.

The scanning trajectory curve is defined as a parametric curve s(t), and its explicit mathematical expression can be expressed in the form of a quintic B-spline:

(18) $$\begin{array}{c} s(t)=\sum_{i=0}^{n}\;P_{i}N_{i,5}(t),t\in [0,\;1] \end{array}$$

$s(t)\in R^{3}$ represents the position vector on the trajectory (the end position of the robot), which is a three-dimensional space vector;

$P_{i}\in R^{3}$: the ith B-spline control point;

$N_{i,5}(t)$ is a quintic (order k=5) B-spline basis function;

t is a dimensionless parameter with a range of [0, 1], which represents the process of the trajectory from the starting point (t=0) to the end point (t=1).

In order to define the smoothness index of the trajectory, the second derivative $s^{\prime \prime }(t)$ (acceleration) of the trajectory is defined as:

(19) $$\begin{array}{c} s^{\prime \prime }(t)=\frac{d^{2}s\left(t\right)}{dt^{2}}=\sum_{i=0}^{n}\;P_{i}N_{i,5}^{\prime \prime }(t),t\in [0,\;1] \end{array}$$

$N_{i,5}^{\prime \prime }(t)$: the second-order derivative function of the quintic B-spline basis function (derived from the Cox–de Boor formula);

$s^{\prime \prime }(t)$ is the acceleration vector of the trajectory point at the parameter t, which represents the curvature of the trajectory curve in space and the intensity of the change.

According to the definition of Cox–de Boor recursive formula, the second derivative of the quintic B-spline basis function is defined as:

Based on the basic definition of zero-order basis function:

(20) $$N_{i,0}(t)=\left\{\begin{array}{cc} 1, & \;t_{i}\leq t<t_{i+1}\\ 0, & \;\mathrm{other} \end{array}\right.$$

Higher-order basis functions are defined by the following recursive formula:

(21) $$\begin{array}{c} N_{i,k}(t)=\frac{t-t_{i}}{t_{i+k}-t_{i}}N_{i,k-1}(t)+\frac{t_{i+k+1}-t}{t_{i+k+1}-t_{i+1}}N_{i+1,k-1}(t) \end{array}$$

The second derivative basis function can be obtained by derivative step by step:

(22) $$\begin{array}{c} N_{i,k}^{\prime \prime }(t)=\frac{k(k-1)}{\left(t_{i+k}-t_{i})(t_{i+k-1}-t_{i}\right)}N_{i,k-2}(t)-2\frac{k(k-1)}{\left(t_{i+k}-t_{i})(t_{i+k}-t_{i+1}\right)}N_{i+1,k-2}(t)+\frac{k(k-1)}{\left(t_{i+k+1}-t_{i+1})(t_{i+k}-t_{i+1}\right)}N_{i+1,k-2}(t) \end{array}$$

k = 5.

Smoothness evaluation function:

In order to evaluate the smoothness of the robotic’s scanning trajectory, the square integral form of the trajectory acceleration is usually used:

(23) $$\begin{array}{c} J_{\mathrm{smooth}}=\int_{0}^{1}∥s^{\prime \prime }(t){∥}^{2}dt=\int_{0}^{1}\left[(\frac{d^{2}x(t)}{dt^{2}}{)}^{2}+(\frac{d^{2}y(t)}{dt^{2}}{)}^{2}+(\frac{d^{2}z(t)}{dt^{2}}{)}^{2}\right]dt \end{array}$$

*x*(*t*), *y*(*t*), *z*(*t*) represent the variations in the three spatial coordinates over time.

In the actual calculation, the above integral is approximated by discretization:

(24) $$\begin{array}{c} J_{\mathrm{smooth}}\approx \sum_{j=1}^{M}∥s^{\prime \prime }\left(t_{j}\right){∥}^{2}\Delta t,t_{j}\in [0,1],j=1,2,\ldots ,M \end{array}$$

M is the number of discrete points. Δt=1/M.

C^3^ continuous local path optimization method.

In order to further improve the efficiency of trajectory optimization, this paper adopts the local path segmentation optimization method. In each local area of the blade surface, the robot trajectory needs to meet the C^3^ continuous condition, which is as follows:

Position continuous: $s\left(t\right)$ continuous;

First derivative (velocity) continuity: $s^{\prime }\left(t\right)$ continuity;

Second derivative continuous (acceleration): $s^{\prime \prime }(t)$ continuous.

The mathematical model of local trajectory optimization is as follows:

(25) $$\begin{array}{c} \begin{array}{cc} \underset{P_{local}}{min}J_{local} & =\int_{t_{a}}^{t_{b}}∥s^{\prime \prime }(t){∥}^{2}dt+\eta \int_{t_{i}}^{t_{i+1}}∥s(t)-p_{surf}(t){∥}^{2}dt\\ & s^{\left(k\right)}(t_{a})=s_{a}^{\left(k\right)},s^{\left(k\right)}(t_{b})=s_{b}^{\left(k\right)},k=0,1,2 \end{array} \end{array}$$

$s^{\left(k\right)}\left(t_{a}\right),\;\;s^{\left(k\right)}(t_{b})$ represent the derivative boundary conditions of the boundary at both ends of the local trajectory (inherited from the trajectory of the previous region), respectively, to ensure that the trajectory can achieve C^3^ continuity at the splicing of each segment.

### 2.6. G^3^ Continuous Trajectory Planning Based on Quintic B-Splines

The continuity and smoothness of the robot trajectory are crucial to the quality of blade detection, the motion performance, and the life of the robot. In order to realize the high-order geometric continuity (G^3^ continuity, i.e., position, velocity, acceleration, and jerk continuity) of the robot trajectory, this section proposes a G^3^ continuous trajectory planning method based on the quintic B-spline curve, and carries out detailed mathematical modeling and derivation.

The robot trajectory curve s(t) for the detection of curved components such as blades is represented as a quintic B-spline form, which is as follows:

(26) $$\begin{array}{c} s(t)=\sum_{i=0}^{n}P_{i}N_{i,5}\left(t\right),t\in [0,1] \end{array}$$

$P_{i}$ is the i-th control point of the B-spline, $P_{i}=[x_{i},y_{i},z_{i}{]}^{T}\in R^{3}$;

$N_{i,5}(t)$ is a quintic B-spline basis function, which is defined by Cox–de Boor recursive formula. The parameter t∈[0,1] is the normalized parameter of the trajectory.

In order to clearly define the continuity characteristics of the trajectory, the explicit definition of each order derivative of the trajectory is further given.

The first derivative (velocity) of the trajectory is defined as:

(27) $$\begin{array}{c} s^{\prime }(t)=\frac{ds(t)}{dt}=\sum_{i=0}^{n}P_{i}N_{i,5}^{\prime }(t) \end{array}$$

The second derivative (acceleration) of the trajectory:

(28) $$\begin{array}{c} s^{\prime \prime }(t)=\sum_{i=0}^{n}P_{i}N_{i,5}^{\prime \prime }(t) \end{array}$$

The third-order derivative of the trajectory:

(29) $$\begin{array}{c} s^{\left(3\right)}(t)=\sum_{i=0}^{n}P_{i}N_{i,5}^{\prime \prime \prime }(t) \end{array}$$

The derivative basis functions are derived step by step by the Cox–de Boor formula.

According to the above recursive formula, the explicit expressions of the quintic B-spline and its derivatives can be obtained in turn, which can be used to construct the robot ultrasonic scanning trajectory for the blade.

(1) Definition of objective function for trajectory smoothness optimization:

In order to realize the optimization of trajectory smoothness, the smoothness objective function is defined as the square integral of trajectory acceleration and jerk:

(30) $$\begin{array}{c} J_{G^{3}}=\alpha \int_{0}^{1}∥s^{\prime \prime }(t){∥}^{2}dt+\beta \int_{0}^{1}∥s^{\left(3\right)}(t){∥}^{2}dt \end{array}$$

Discrete processing in the actual calculation:

(31) $$\begin{array}{c} J_{G^{3}}\approx \alpha \sum_{j=1}^{M}∥s^{\prime \prime }(t_{j}){∥}^{2}\Delta t+\beta \sum_{j=1}^{M}∥s^{\left(3\right)}(t_{j}){∥}^{2}\Delta t \end{array}$$

$s^{\prime \prime }(t_{j})$, $s^{\left(3\right)}(t_{j})$ can be obtained by the above formula;

α,  β are the integral weights of acceleration and jerk, Δt=1/M.

(2) Definition of constraints:

In order to ensure that the trajectory of the ultrasonic beam incident point always fits the blade surface, the distance constraint between the trajectory and the blade surface is defined:

(32) $$\begin{array}{c} d(s(t),p_{surf}(t))=∥s(t)-p_{surf}(t)∥\leq \delta ,t\in [0,1] \end{array}$$

$p_{surf}(t)$: The projection point of the trajectory point on the blade surface;

δ: The allowable maximum distance threshold between the robot trajectory and the blade surface.

Combining the optimization objectives and constraints defined above, an overall mathematical optimization model is constructed:

(33) $$\begin{array}{cc} \underset{P}{min} & J_{G^{3}}(P)=\alpha \sum_{j=1}^{M}∥s^{\prime \prime }(t_{j}){∥}^{2}\Delta t+\beta \sum_{j=1}^{M}∥s^{\left(3\right)}(t_{j}){∥}^{2}\Delta t\\ & ∥s(t_{j})-p_{surf}(t_{j})∥\leq \delta ,j=1,\ldots ,M\\ & s(t)\in \Omega_{B},t\in [0,\;1] \end{array}$$

P is the control point to be optimized;

$\Omega_{B}$ is the outer envelope workspace constraint of the robot trajectory;

δ is the maximum allowable deviation;

M is the number of discrete sampling points.

## 3. Experiment Results and Discussion

**Figure 3:**
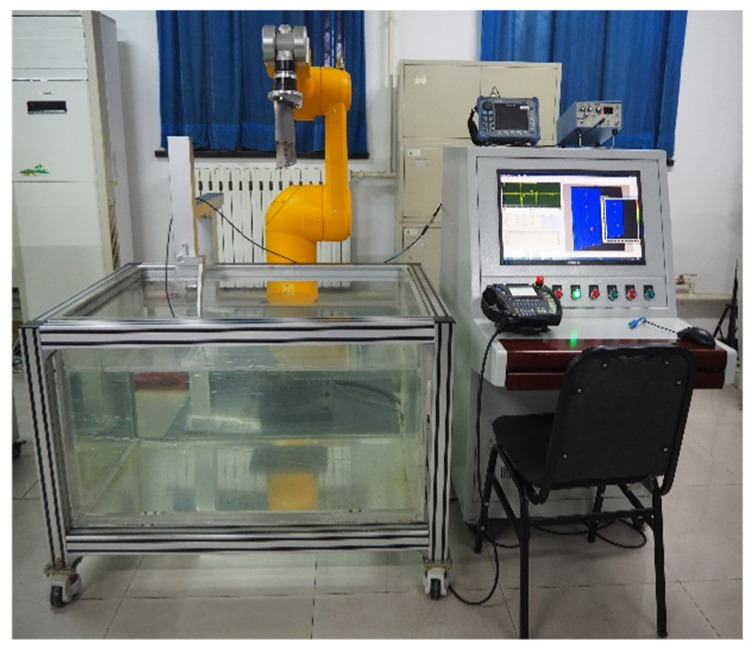
Scheme of an ultrasonic nondestructive testing system for robotics.

**Figure 4:**
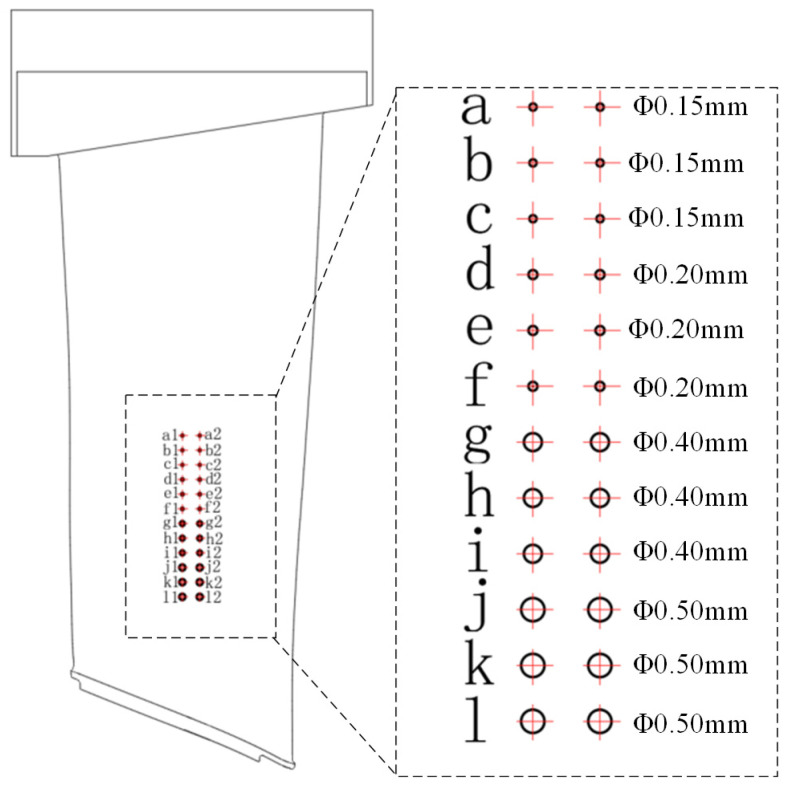
Artificial flat bottom hole and crack defects of the blade body.

In the present study, the system depicted in Figure 3 was developed for the assessment of engine blades. Figure 4 shows the artificial flat-bottom hole defect that was prefabricated in the blade body. The blade being examined is mounted on the end effector of a Stäubli industrial robot, enabling its movement relative to a stationary ultrasonic transducer for thorough sweeping inspection. A comprehensive list of the system components is provided in Table 1.

### 3.1. Experimental Verification and Comparative Analysis

**Figure 5:**
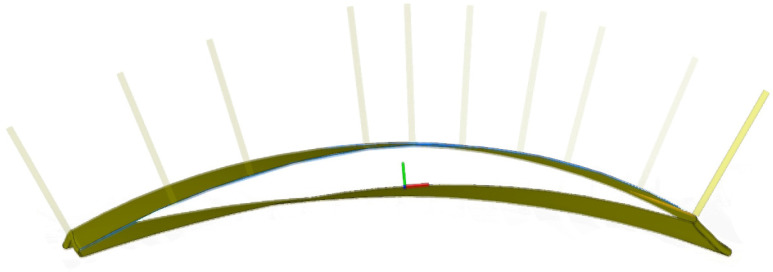
Complex surface trajectory of an aero-engine blade.

The present study evaluates the effectiveness of the proposed robotic ultrasonic testing methodology for identifying defects in complex, curved-surface components exhibiting irregular curvature—exemplified by aerospace blades. Particular emphasis is placed on how the smoothness of the scanning trajectory influences the robot’s vibration characteristics and the accuracy of defect detection. To emulate representative flaw geometries encountered in blade inspections, flat-bottomed holes of precisely controlled diameters and depths were machined into the specimen surface (see Figure 4). To validate the effectiveness of the trajectory-optimization algorithm, scanning trajectory planning was carried out on the measured blade (see Figure 5), after which two sets of robotic scanning paths, before and after optimization, were selected for comparative analysis. These paths were assessed in terms of their acceleration-profile characteristics, quantitative smoothness indices, and resulting ultrasonic C-scan images.

**Figure 6:**
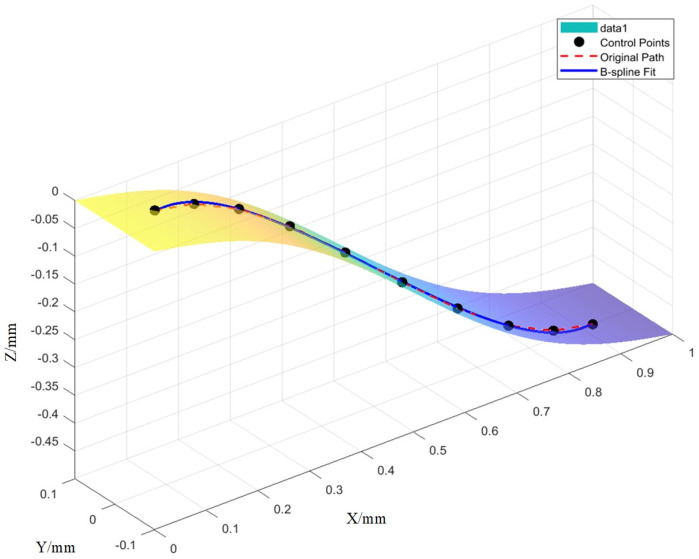
Comparison of scanning paths before and after optimization.

Figure 6 presents a comparative overlay of the robot trajectories before and after optimization. The pre-optimization trajectory exhibits pronounced angular discontinuities (C^1^ discontinuities) at the polyline vertices, which induce abrupt variations in both velocity and acceleration during motion. In contrast, the optimized trajectory is globally C^2^-continuous: curvature transitions occur smoothly without any perceptible jitter or abrupt changes. Examination of the acceleration time history further reveals that the optimized profile is markedly smoother peak accelerations at each sampling instant that are substantially diminished, high-frequency oscillatory components are effectively attenuated, and the overall waveform evolves as a low-amplitude, low-frequency, slowly varying process.

To quantify the improvement afforded by the optimization, acceleration histories were extracted at ten equispaced points along both the original and the optimized trajectories. These datasets were then subjected to systematic comparative analysis, the results of which are summarized in Table 2.

**Figure 7:**
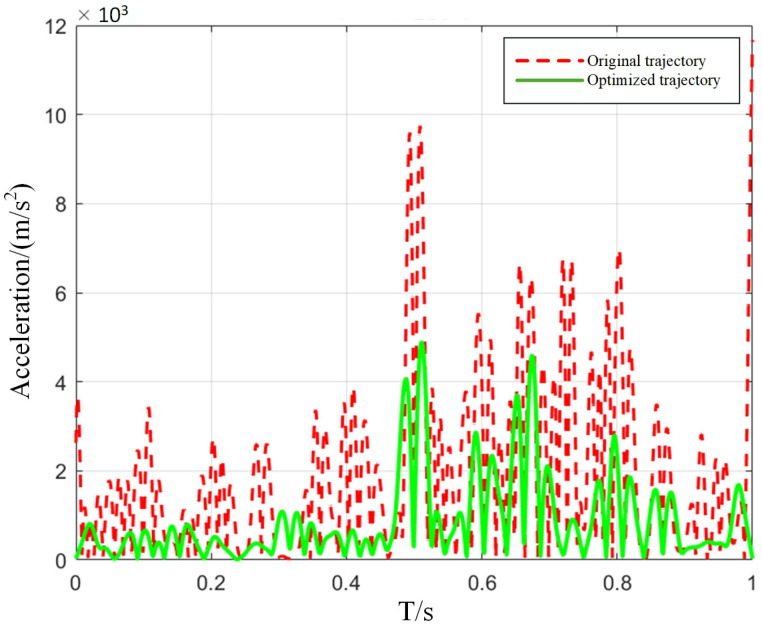
Comparison of trajectory acceleration before and after optimization.

As illustrated in Figure 7, the acceleration profile along the entire trajectory exhibits markedly fewer peaks following optimization, with the peak amplitudes substantially attenuated—thereby demonstrating significantly enhanced stability.

The improved proportion calculation method in the table:

(34) $$Improvement\;ratio\;(\%)=\frac{Original\;acceleration-Optimized\;acceleration}{Original\;acceleration}\times 100\%$$

The results of the data analysis indicate that, following trajectory optimization, the manipulator’s acceleration fluctuations during motion are reduced by approximately 68% on average. This diminution of dynamic oscillations markedly mitigates vibration during the scanning process, thereby enhancing the stability and measurement accuracy of blade inspection. To further quantify improvements in trajectory smoothness, curvature is adopted as the evaluation metric, calculated according to the following expression:

(35) $$k(t)=\frac{\left|r^{\prime }(t)\times r^{\prime \prime }(t)\right|}{|r^{\prime }(t){|}^{3}}$$

**Figure 8:**
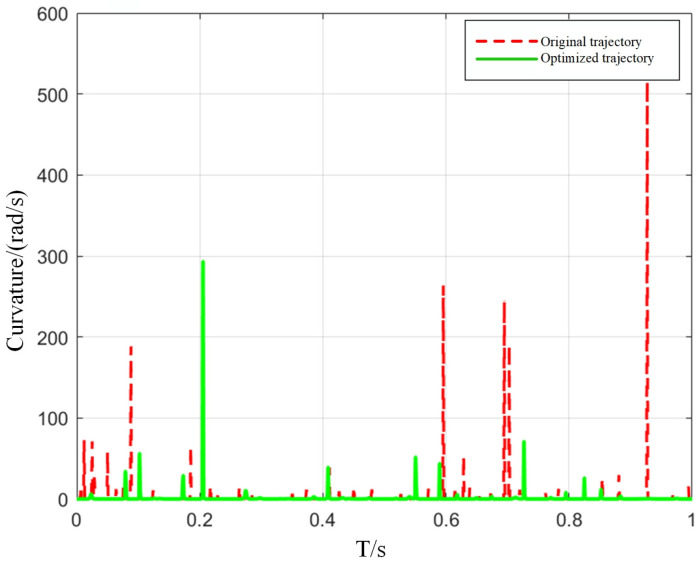
Comparison of trajectory curvature before and after optimization.

$r^{\prime }(t)$, $r^{\prime \prime }(t)$ are the first and second derivatives of the path, respectively. The improvement of curvature is shown in Figure 8:

Figure 8 shows the comparative analysis of the curvature change of the whole trajectory interval. The original trajectory shows obvious fluctuations, and the maximum curvature change exceeds 500 rad/s. In contrast, the optimized trajectory shows a more stable curvature, which is usually kept below 300 rad/s, thereby significantly reducing severe local bending.

The research findings demonstrate that the proposed trajectory optimization method significantly enhances the continuity and smoothness of the scanning path. This improvement effectively mitigates the vibrations generated by the robot during the blade detection process, thereby meeting the stringent stability and accuracy requirements necessary for practical engineering applications.

### 3.2. Comparison of Robot Scanning Speed Changes

**Figure 9:**
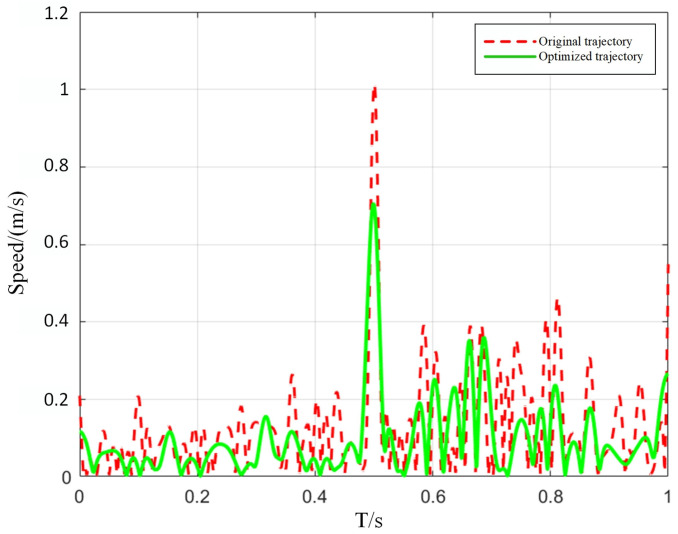
Comparison of scanning trajectory speed before and after optimization.

The enhancement of trajectory smoothness not only theoretically optimizes the continuity of path planning but also significantly improves the stability of the robot’s running speed during actual motion. To further assess this effect, this study compares the velocity time-history data of the robot along the blade scanning path before and after optimization under identical working conditions. The velocity time-history overlay comparison, as shown in Figure 9, visually illustrates the aforementioned quantitative results: the optimized velocity curve exhibits a more uniform fluctuation pattern, and the velocity variation between sampling points becomes smoother. These findings comprehensively demonstrate that path smoothing not only improves the motion coherence of the manipulator but also positively influences its dynamic response characteristics, thereby providing a solid operational foundation for high-precision blade detection tasks.

As shown in the figure, the speed standard deviation decreases from 0.305 m/s before optimization to 0.115 m/s after optimization 62.3% reduction in variability. Concurrently, the speed range contracts from 1 m/s to 0.7 m/s, thereby substantiating that the optimization significantly enhances the manipulator’s motion stability.

In summary, trajectory-optimized robot motion planning confers notable benefits for ultrasonic blade-scanning operations. First, the optimized trajectories exhibit superior spatial and temporal continuity, effectively attenuating high-frequency vibrations during robot movement. Second, the amplitude of acceleration fluctuations is markedly diminished, ensuring more consistent coupling between the ultrasonic transducer and the blade surface, which in turn enhances the signal-to-noise ratio and improves imaging fidelity. Moreover, the robot’s smoother dynamic response mitigates inertial impacts throughout the scanning process, thereby extending system longevity and bolstering overall reliability.

### 3.3. C-Scan Imaging of the Leaf Body

**Figure 10:**
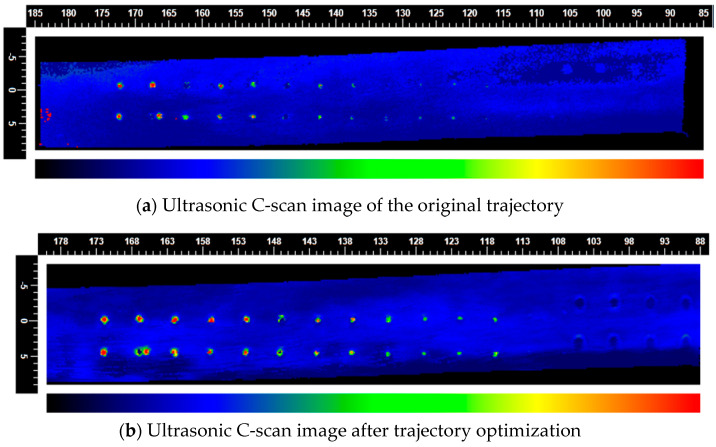
Ultrasonic C-scan image of engine blade.

Experimental results demonstrate that twenty-four flat-bottomed hole defects (diameter range: 0.15–0.50 mm), arranged within the designated inspection region, yield high-contrast ultrasonic C-scan images, confirming that the selected 20 MHz transducer and 250 MHz sampling-rate data-acquisition system afford adequate spatial resolution and signal-to-noise performance. By contrast, when employing the unoptimized scanning trajectory (Figure 10a), excessive vibration between the robot’s end effector and the workpiece leads to echo-signal distortion and overlap. This not only degrades defect contrast but also exacerbates artifacts, thereby markedly diminishing the detectability of small defects—particularly those on the order of 0.15 mm.

Following the implementation of trajectory smoothing and acceleration-profile optimization, vibration amplitudes during robot motion are effectively attenuated, resulting in substantially enhanced contrast and clarity in the ultrasonic C-scan images. In particular, the accuracy of small-defect localization and dimensional assessment is improved; the optimized trajectory preserves the phase and amplitude integrity of the echo waveforms, thus elevating both image resolution and measurement precision.

## 4. Conclusions

The present study introduces a G^3^-continuous trajectory optimization framework for robotic ultrasonic scanning of complex free-form surfaces, underpinned by quintic B-spline curve modeling. By formulating the robot’s end-effector path as a parametric quintic B-spline, we derive closed-form expressions for position, velocity, acceleration, and jerk, thereby guaranteeing C^3^ (G^3^) continuity throughout the trajectory. This high-order geometric smoothness effectively mitigates abrupt motion changes and suppresses oscillatory behavior—critical factors when negotiating regions of high curvature or irregular morphology (e.g., turbine blades).

To balance motion stability against surface-conformity requirements, we construct a composite objective function comprising two key terms: (1) the integrated square of the second and third derivatives of the trajectory (acceleration and jerk), which serves as a smoothness regularizer; and (2) the mean square error between the B-spline path and the target workpiece surface, which quantifies fitting accuracy. A numerical optimization scheme is then employed to minimize this composite cost, yielding an optimal set of control points for the quintic B-spline.

Simulation and experimental results validate the effectiveness of the proposed method. It significantly reduces trajectory mutations and oscillations, successfully achieving efficient and accurate automated ultrasonic testing. This approach offers a reliable trajectory planning strategy for online nondestructive testing of complex curved workpieces, demonstrating significant application potential and practical value.

The proposed method may encounter challenges in maintaining real-time performance when subjected to unpredictable surface variations. Additionally, it may not be well-suited for irregular or discontinuous geometries, where the assumptions underlying the approach may fail to deliver accurate results.

## Figures and Tables

**Table 1:** Main components of the robot-assisted UT system.

Apparatus	Brand	Model	Property
Robot	Staubli	TX90L	Repeatability: 0.035 mm
Ultrasonic Pulser/Receiver	Olympus	5077PR	Receiving bandwidth: 1 KHz–25 MHz
Ultrasonic probe	Olympus	V319	20 MHz
A/D Acquisition Card	Acquisition Logic	AL12200	sample rate: 250 MHz
Container	Non-standard	Non-standard	Volume: 500 L

**Table 2:** Comparison data of path acceleration before and after optimization (m/s^2^).

Sampling Point	Original Acceleration	Optimized Acceleration	Improvement Ratio (%)
1	250	85	66.0%
2	310	102	67.1%
3	460	118	74.3%
4	510	133	73.9%
5	720	205	71.5%
6	850	265	68.8%
7	910	300	67.0%
8	980	340	65.3%
9	1030	370	64.1%
10	1050	390	62.9%

## Data Availability

Data is contained within the article. The data presented in this study are available in Experiment Results and Discussion.

## References

[1] Morozov M., Pierce S.G., MacLeod C.N., Mineo C., Summan R. (2018). Off-line Scan Path Planning for Robotic NDT. Measurement.

[2] Ajman M.A.A., Abdullah E.J. (2024). Design of a Robotic Arm for Inspecting Curved Surface in Aerospace Non-Destructive Testing. J. Aeronaut. Astronaut. Aviat..

[3] Zhang H., Xu C., Xiao D. (2018). Crack assessment of wheel hubs via an ultrasonic transducer and industrial robot. Sensors.

[4] Mineo C., Pierce S.G., Nicholson P.I., Cooper I. (2016). Robotic path planning for non-destructive testing—A custom MATLAB toolbox approach. Robot. Comput. Integr. Manuf..

[5] Morozov M., Mineo C., Pierce S.G., Nicholson P.I., Cooper I. Computer-Aided Tool Path Generation for Robotic Non-Destructive Inspection. Proceedings of the Conference of the British Institute of Non-Destructive Testing 2013.

[6] Secanellas S.A., León I.G., Parrilla M., Acebes M., Ibáñez A., Jiménez H.d.M., Martínez-Graullera Ó., de Pablos A.Á., Hernández M.G., Velayos J.J.A. (2023). Methodology for the Generation of High-Quality Ultrasonic Images of Complex Geometry Pieces Using Industrial Robots. Sensors.

[7] Xiao Z., You Y., Xu C.G., Xiao D.G. (2018). Profile tracking with ultrasonic alignment for automatic non-destructive testing of complex structures. Robot. Comput. Integr. Manuf..

[8] Kovarikova Z., Duchon F., Porubsky M., Trebula M., Chovanec L., Salat E., Rakyta M. (2025). Robotic Ultrasound Diagnostic System for Non-Destructive Testing in Highly Variable Production. Electronics.

[9] Iakovleva E., Roué D., Brédif P. (2024). Automatic image alignment and stitching for ultrasound-based robotic inspection of complex geometry components. NDT E Int..

[10] Mineo C., Pierce S.G., Wright B., Copper I., Nicholson P.I. (2015). PAUT inspection of complex-shaped composite materials through six DOFs robotic manipulators. Insight Non-Destr. Test. Cond. Monit..

[11] Mineo C., MacLeod C., Morozov M., Pierce S.G., Lardner T., Summan R., Powell J., McCubbin P., McCubbin C., Munro G. Fast ultrasonic phased array inspection of complex geometries delivered through robotic manipulators and high speed data acquisition instrumentation. Proceedings of the 2016 IEEE International Ultrasonics Symposium (IUS).

[12] Zhou B., Tian T., Zhu G., Zhao J., Liu D. (2022). An ultrasonic testing method for wall thickness of turbine blades. Measurement.

[13] Yang J., Yuen A. (2017). An analytical local corner smoothing algorithm for five-axis CNC machining. Int. J. Mach. Tools Manuf..

[14] Tulsyan S., Altintas Y. (2015). Local toolpath smoothing for five-axis machine tools. Int. J. Mach. Tools Manuf..

[15] Leng H., Wu Y., Pan X. Research on flexible acceleration and deceleration method of NC system. Proceedings of the 2006 International Technology and Innovation Conference (ITIC 2006).

[16] Ye P., Shi C., Yang K., Lv Q. (2008). Interpolation of continuous micro line segment trajectories based on look-ahead algorithm in high-speed machining. Int. J. Adv. Manuf. Technol..

[17] Shi C., Ye P. (2011). The look-ahead function-based interpolation algorithm for continuous micro-line trajectories. Int. J. Adv. Manuf. Technol..

[18] Shi J., Bi Q.Z., Wang Y.H., Liu G. (2014). Development of Real-Time Look-Ahead Methodology Based on Quintic PH Curve with G2 Con-tinuity for High-Speed Machining. Appl. Mech. Mater..

[19] Huang N., Jin Y., Bi Q., Wang Y. (2015). Integrated post-processor for 5-axis machine tools with geometric errors compensation. Int. J. Mach. Tools Manuf..

[20] Erkorkmaz K., Altintas Y. (2001). High speed CNC system design. Part I: Jerk limited trajectory generation and quintic spline interpola-tion. Int. J. Mach. Tools Manuf..

[21] Bi Q., Wang Y., Zhu L., Ding H. An Algorithm to Generate Compact Dual NURBS Tool Path with Equal Distance for 5-Axis NC Ma-chining. Proceedings of the Intelligent Robotics and Applications—Third International Conference, ICIRA 2010.

[22] Bi Q., Wang Y., Zhu L., Ding H. A Practical Continuous-Curvature Bézier Transition Algorithm for High-Speed Machining of Linear Tool Path. Proceedings of the Intelligent Robotics and Applications.

[23] Peng J., Ding Y., Zhang G., Ding H. (2020). Smoothness-oriented path optimization for robotic milling processes. Sci. China Technol. Sci..

[24] Walton D.J., Meek D.S. (2009). G2 blends of linear segments with cubics and Pythagorean-hodograph quintics. Int. J. Comput. Math..

[25] Zhao H., Zhu L.M., Ding H. (2013). A real-time look-ahead interpolation methodology with curvature-continuous B-spline transition scheme for CNC machining of short line segments. Int. J. Mach. Tools Manuf..

[26] Beudaert X., Lavernhe S., Tournier C. (2013). 5-axis local corner rounding of linear tool path discontinuities. Int. J. Mach. Tools Manuf..

[27] Shi J., Bi Q.Z., Zhu L.M., Wang Y.H. (2015). Corner rounding of linear five-axis tool path by dual PH curves blending. Int. J. Mach. Tools Manuf..

[28] Peng J., Huang P., Ding Y., Ding H. (2021). An Analytical Method for Decoupled Local Smoothing of Linear Paths in Industrial Robots. Robot. Comput.-Integr. Manuf..

[29] Zhao X., Wang H., Ding H. (2021). Quaternion-based smooth trajectory optimization for robotic inspection tasks on free-form surfaces. Robot. Auton. Syst..

[30] Siciliano B., Sciavicco L., Villani L., Oriolo G. (2010). Robotics: Modelling, Planning and Control.

